# Theoretical and Experimental Comparison of Different Formats of Immunochromatographic Serodiagnostics

**DOI:** 10.3390/s18010036

**Published:** 2017-12-25

**Authors:** Dmitriy V. Sotnikov, Anatoly V. Zherdev, Boris B. Dzantiev

**Affiliations:** A.N. Bach Institute of Biochemistry, Research Center of Biotechnology of the Russian Academy of Sciences, Leninsky Prospect 33, Moscow 119071, Russia; sotnikov-d-i@mail.ru (D.V.S.); zherdev@inbi.ras.ru (A.V.Z.)

**Keywords:** lateral flow immunoassay, detection of antibodies, mathematic simulation, tuberculosis

## Abstract

In this study, a comparative theoretical and experimental analysis of two immuno-chromatographic serodiagnostics schemes, which differ in the immobilization of immunoreagents and the order of the formation of immune complexes, is performed. Based on the theoretical models, the assays are characterized to determine which scheme has a higher quantity of the detected complex and thus ensures the sensitivity of the analysis. The results show that for the effective detection of low-affinity antibodies, the scheme involving the immobilization of the antigen on gold nanoparticles and the antibody-binding protein on the test strip was more sensitive than the predominantly used scheme, which inverts the immunoreagents’ locations. The theoretical predictions were confirmed by the experimental testing of sera collected from tuberculosis patients.

## 1. Introduction

Immunochemical test systems are widely used in medical and veterinary diagnostics [1]. Serodiagnostics is a form of immunoassay used to detect antibodies specific to the infectious agent in the blood. These antibodies are generated in response to the presence of an infectious agent in the body, and detecting them is often more informative than detecting the pathogen [2,3]. Immunochromatographic serodiagnostics are conducted using lateral flow test strips, which are also especially promising for mass use. Testing can be conducted outside the laboratory because it does not require equipment or highly qualified personnel. Importantly, the results can be obtained in 10–15 min with this method [4,5,6,7]. Because of their rapidity, methodological simplicity and low cost, immunochromatographic assays (ICA) are widely used in medical and veterinary diagnostics. In addition, the above advantages make serodiagnostic ICA an effective tool for evaluating the results of immunization of humans and experimental animals.

**Figure 1:**
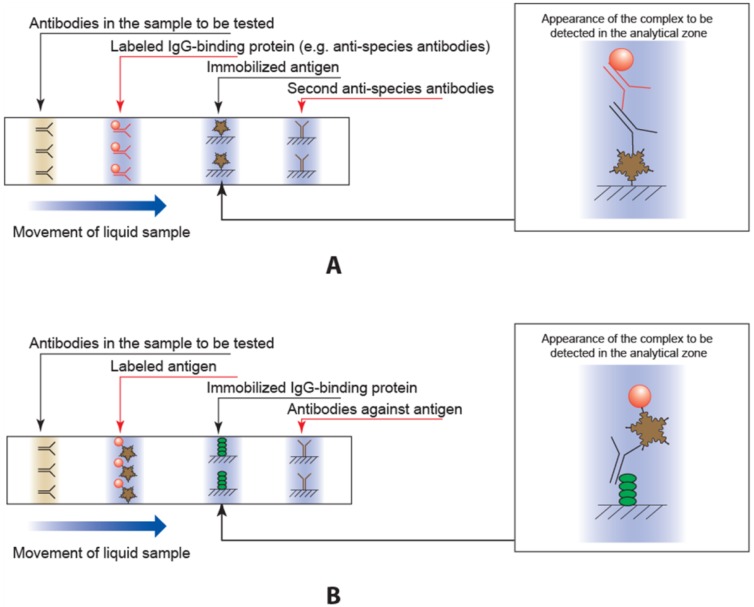
Traditional scheme A (**A**) and alternate scheme B (**B**) of immunochromatographic serodiagnosis.

The traditional scheme for immunochromatographic serodiagnosis (i.e., scheme A) is shown in Figure 1A. In this scheme, a marker (usually gold nanoparticles) is conjugated with an immunoglobulin-binding protein (e.g., anti-species antibodies, staphylococcal protein A, or streptococcal protein G). A sample of blood or serum then flows along the membranes of the test strip. In the first stage, all immunoglobulins in the sample interact with the conjugate. Next, the liquid reaches the zone of the strip with the immobilized antigen (i.e., the analytic zone). The conjugate interacts with the antigen molecules, resulting in the formation of a colored complex containing gold nanoparticles, immunoglobulin-binding protein molecules, immunoglobulins from the sample, and immobilized antigen. The given scheme is used in the most of lateral flow tests described in the literature [8,9,10,11,12], as well as in the most of the manufactured serodiagnosis tests such as the products of the companies AmeriTek (Everett, WA, USA; www.ameritek.org), Dr. Fooke Laboratories (Neuss, Germany; www.fooke-labs.de), Human (Wiesbaden, Germany; www.human.de), Standard Diagnostics (Yongin-si, Korea; www.standardia.com), Vedalab (Alençon, France; www.vedalab.com), etc.

Serodiagnostic ICA may be realized in other formats that differ in the order of detectable complex formation and, accordingly, in the composition of the colored complex in the analytic zone [13,14,15,16]. The most popular alternate format of serodiagnostic ICA is a scheme (scheme B) in which the antigen molecules are conjugated to gold nanoparticles, and the immunoglobulin-binding protein is immobilized on the test strip (Figure 1B). In this ICA, specific immunoglobulins first interact with the antigen. The gold nanoparticles conjugate, form complexes, then migrate to the analytic zone and bind there. Both kinds of test strips also contain a second control zone with immobilized antibodies (anti-species ones in scheme A and antibodies against the antigen for scheme B) to control the strips’ functionality.

Sotnikov et al. [16] showed that in some cases, scheme B allowed increased diagnostic sensitivity of the analysis. However, it is not known whether scheme B is always characterized by a higher sensitivity than scheme AA, or if this is true only under certain conditions. Moreover, the causes of the change in sensitivity when an alternate scheme is used remain unexplained.

To address these issues, the present study simulates the processes of immune interactions in two ICA schemes and compares their characteristics. In a recent study [17], a mathematical model of a serodiagnostic ICA with the traditional (i.e., A) scheme was proposed. Scheme B has not been previously described using mathematical tools. The development and analysis of this model are provided for the first time in this article. In addition, the obtained model is compared with the model of the traditional scheme A, and the advantages of each analytical scheme are then justified. The potential of scheme B to increase sensitivity is confirmed experimentally using the serodiagnosis of one of the most significant infectious diseases: human pulmonary tuberculosis.

## 2. Materials and Methods

### 2.1. Reagents

3,3′,5,5′-Tetramethylbenzidine (TMB), chloroauric acid, bovine serum albumin (BSA), and sodium azide were obtained from Sigma-Aldrich (St. Louis, MO, USA). Recombinant 38-kDa antigen (Rv0934) of *Mycobacterium tuberculosis* was obtained from Arista Biologicals (product code AGMTB-0220, Allentown, PA, USA), and recombinant staphylococcal protein A was obtained from Imtek (Moscow, Russia). All salts and other additional reactants were of analytical or reagent grade. The Simplicity purification system (Millipore, Bedford, MA, USA) was used for water deionization.

Serum samples from healthy donors were supplied by the State Scientific Center, Institute of Immunology (Moscow, Russia). Serum samples from persons with a confirmed case of pulmonary tuberculosis were supplied by Dr. V. G. Avdienko, Central Tuberculosis Research Institute (Moscow, Russia). In all the serum samples, the presence of anti-mycobacterial antibodies was confirmed by quantitative immunoenzyme assay (see below).

### 2.2. Immunoenzyme Assay of Specific Antibodies in Serum Samples

Antigen Rv0934 was adsorbed in a 96-well Greiner microplate (100 μL per well) for 16 h at 4 °C from a 1 μg/mL solution in 50 mM Na-carbonate buffer (50 mM, pH 9.6). The wells were washed with a K-phosphate buffer (50 mM, pH 7.4), with 0.1 M NaCl and 0.05% detergent Triton X-100 (PBST). Then, of sera 100-fold diluted with PBST was added (100 μL per well) and incubated for 1 h at 37 °C. The microplate was washed, and monoclonal antibodies against human IgG labeled with horseradish peroxidase were added (160 ng/mL, 100 μL in PBST) for a 1-h incubation at 37 °C. After washing, the activity of the bound peroxidase was tested by the addition of 0.4 mM TMB in an Na-citrate buffer (100 mM, pH 4.0), with 0.006% H_2_O_2_ (100 μL per well), incubated for 15 min, and measured for its OD_450_ values (optical density at 450 nm).

### 2.3. Synthesis of Gold Nanoparticles

The Frens method [18] was used for the synthesis: 0.2 mL of 5% chloroauric acid was added to 97.5 mL of water and heated to boiling. After this, 1.5 mL of 1% sodium citrate was added. The reactants were boiled for 25 min and then cooled.

Size of the obtained gold nanoparticles was characterized using CX-100 transmission electron microscope (Jeol, Akishima-shi, Japan) and Image Tool software (University of Texas Health Science Center, San Antonio, TX, USA) as described in [16]. An average diameter of the particles was 28 ± 3 nm.

### 2.4. Conjugation of Protein A and Antigen Rv0934 with Gold Nanoparticles

Firstly, the flocculation curves were obtained for mixtures of the mycobacterial antigen Rv0934 and the staphylococcal protein A with gold nanoparticles as described in [19]. The protein solutions of different concentrations were added to gold nanoparticles preparation, 10% NaCl was then added and OD_590_ of the mixture was measured after 10-min incubation at room temperature. The plateaus of the obtained concentration dependences are considered as zones of the saturation of gold nanoparticles surface by immobilized antibodies. The given saturations were reached for the concentrations equal to 8 µg/mL for the antigen Rv0934 and 20 µg/mL for the protein A, and the given concentrations were chosen for the conjugation.

The antigen Rv0934 and the protein A were dialyzed against an Na-carbonate buffer (10 mM, pH 9.0) and diluted to the concentrations indicated above. The pH value of the gold nanoparticle preparation with OD_520_ equal to 1.0 was shifted to 9.0 using a 0.1 M К_2_СО_3_ and were then added to the antigen Rv0934 and the protein A solutions. The mixture was then stirred for 10 min, and a BSA was added to the final concentration of 0.25%. Finally, the gold nanoparticles with the bound proteins were separated by centrifugation (8000 g, 20 min) and redissolved in PBS with 0.25% BSA. The conjugates were stored at 4 °C with the addition of sodium azide (0.05%) for long-term storage.

### 2.5. Application of Reagents onto Immunochromatographic Membranes

All membranes and pads were obtained from Advanced Microdevices, Ambala Cantt, India. An IsoFlow dispenser (Imagene Technology, Hanover, NH, USA) was used for the reagents application.

In the case of scheme A, the analytic zone was formed by applying the antigen Rv0934 of *M. tuberculosis* water solution (1.0 mg/mL) onto the nitrocellulose membrane CNPH90, 2 µL of the antigen solution per 1 cm. The conjugate of protein A and gold nanoparticles was applied to the conjugate release pad PT-R5 at a dilution corresponding to OD_520_ = 10.0 in a volume of 11 µL per 1 cm.

In the case of scheme B, the analytic zone was formed by applying the protein А onto the nitrocellulose membrane CNPH90, 2 µL of its solution (10 mg/mL in K-phosphate buffer (50 mM, рН 7.4)) was applied per 1 cm. The conjugate of antigen Rv0934 and gold nanoparticles was applied to the conjugate release pad PT-R5 at a dilution corresponding to OD_520_ = 2.0 in a volume of 8 µL per 1 cm.

### 2.6. Preparation of Immunochromatographic Test Strips

The membranes with the applied reagents were air dried for one day. The test strips were prepared using the nitrocellulose membrane, conjugate release pad, a sample pad FR1(0.6), and a final adsorbent pad, AP045 as proposed in [17]. The multimembrane composite was assembled and then cut using an Index Cutter-1 automated guillotine cutter (A-Point Technologies, Gibbstown, NJ, USA) into strips 3.5 mm wide. The strips were packed in laminated aluminum foil bags using a FR-900 conveyor (Dingli Packing Machinery, Wenzhou, China) with added silica gel as the desiccant. The preparation of the test strips was carried out in a room with relative humidity <30%. The manufactured strips were stored at room temperature.

### 2.7. Immunochromatographic Serodiagnostic Assay

One drop of serum to be tested and three drops of PBS containing 1% Tween-20 were added in an Eppendorf tube. Then, a strip was vertically placed in the tube for 10 min. The obtained results were inspected visually and/or quantified using a Reflekom portable photometric analyzer (Synteco, Moscow, Russia).

## 3. Results and Discussion

### 3.1. Designations and Consumptions in Scheme B Modeling

The following designations were used for the components of the system and the parameters of the reactions:

A—immunoglobulins in the sample
*P*—active molecules of the antigen immobilized on gold nanoparticles
*R*—active molecules of immunoglobulin-binding protein immobilized in the analytic zone
*AP*—complex of immunoglobulin with an immunoglobulin-binding protein that is immobilized on a gold nanoparticle surface
*AR*—complex of immunoglobulins with an antigen in the analytic zone
*APR*—complex of specific immunoglobulins with gold conjugate and an antigen in the analytic zone
*К_ai_*—equilibrium association constant of the *i*-th reaction
*k_ai_*—kinetic association constant of the *i*-th reaction
*k_di_*—kinetic dissociation constant of the *i*-th reaction
*t*—time of reaction from the beginning of the sample’s contact with the gold conjugate
*t*_1_—time of reaction in the analytic zone
*x*—proportion of specific immunoglobulins in the total immunoglobulins pool

As the liquid moved along the test strip, the immunoglobulins first interacted with the gold nanoparticles conjugate. This reaction can be expressed by the following equation:

(1) $$A+P\overset{k_{a1},\;k_{d1}}{↔}AP$$

After the liquid reached the analytic zone, it further reacted with the immobilized antigen, which can be represented by the following equations:

(2) $$A+R\overset{k_{a2},\;k_{d2}}{↔}AR$$

(3) $$AP+R\overset{k_{a3},\;k_{d3}}{↔}APR$$

(4) $$P+AR\overset{k_{a4},\;k_{d4}}{↔}APR$$

**Figure 2:**
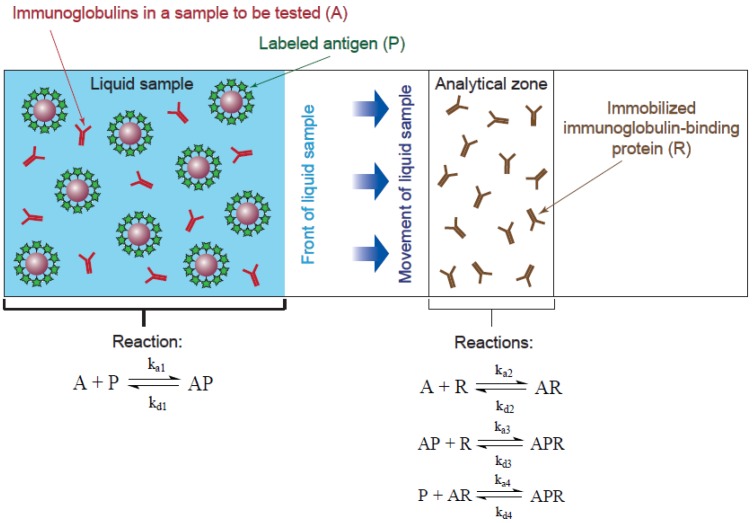
Simplified diagram of the ICA system for serodiagnostics according to the B scheme used to construct the model.

The diagram of the test strip that accords with the given statements is shown in Figure 2.

The following simplifications were used to construct the model for the B scheme:

1. The flow of the liquid sample along the test strip occurred evenly.
2. Two stages of the analysis were considered: in Stage 1, the conjugate of antigen and gold nanoparticle interacted with the immunoglobulins in the sample until the analytic zone was reached (Reaction (1)); in Stage 2, the immunoglobulins interacted with the immunoglobulin-binding protein and the antigen–gold nanoparticle conjugate in the analytic zone (Reactions (2) and (3)).
3. Components *A* and *P* were uniformly distributed in the reaction volume of the liquid sample, and reagent *R* was uniformly distributed within the analytic zone (Figure 2). The boundary of the analytic zone was permeable for reagents *A* and *P* and their complex, but it was impermeable to *R* and its complexes *AR* and *APR*. The uniform distribution of the components within the reaction volumes meant the interaction processes were homogeneous.
4. Polyvalent interactions were not taken into account.
5. Subpopulations of antibodies with different affinities to the antigen were not taken into account.
6. The antibodies did not change the constant of their binding to the immunoglobulin-binding protein after interaction with the antigen or to the antigen after interaction with the immunoglobulin-binding protein; *k_a_*_1_ ≈ *k_a_*_4_; *k_a_*_2_ ≈ *k_a_*_3_.

The signal in ICA was proportional to the concentration of the *APR* complex. Therefore, the main modeling task was to find the kinetic dependence of the changes in the *APR*. The distribution of the *APR* complex in the volume of the analytic zone could be nonuniform. Therefore, for accuracy, the concentration of the complex on the left (Figure 2) boundary of the analytic zone was calculated. To calculate the kinetic dependence of *APR*, it was necessary to derive the equations for changes in *AP*, *R*, and *AR*.

### 3.2. Consideration of the Model: Stage 1

For most antibodies, the kinetic dissociation constant of their binding with the target antigen is characterized by the values *k_d_* < 10^4^ M^−1^·s^−1^, as the analysis of 1450 monoclonal antibodies by Landry et al. [20] confirmed. If the dissociation is irreversible, then the fraction of the *AP* complex decaying per unit time (*D*) can be calculated based on the following equation [21]:

(5) $$D=1-e^{-k_{d}t_{1}}$$

This equation indicates that for *k_d_* < 10^4^ M^−1^·s^−1^, less than 7% of the complex dissociates in 10 min. Therefore, its dissociation during the assay can be neglected, and the *AP* complex formation can be considered an irreversible process.

Thus, the binding of the immunoglobulins with the antigen–gold nanoparticle conjugate was in accordance with an irreversible bimolecular reaction. In the case of scheme B, only specific antibodies (their portion in the total content of immunoglobulins equaled *x*) participated in this reaction. Therefore, the kinetics of the *AP* complex formation were described by the equation of an irreversible bimolecular reaction between the *P* and *A* compounds, in which the initial concentration of immunoglobulins in sample [*A*]_0_ was replaced by *x·*[*A*]_0_:

(6) $$\left[AP\right]=\frac{x\left[A_{0}\right]\left[P_{0}\right]\left(e^{tk_{a1}\left(\left[P_{0}\right]-\left[A_{0}\right]x\right)}-1\right)}{\left[P_{0}\right]e^{{tk}_{a1}\left(\left[P_{0}\right]-\left[A_{0}\right]x\right)}-\left[A_{0}\right]x}$$

the subscript 0 here and in the equations below denotes the initial concentrations.

Using the characteristic values of the kinetic constant of antigen–antibody association (*k_a_*_1_) in the 10^4^ and 10^6^ M^−1^·s^−1^ range and the concentrations of reagents typical in immunochromatographic serodiagnosis, kinetic curves of the formation of the *AP* complex were constructed. Note that the liquid sample movement time to the analytic zone usually did not exceed 1 min. The obtained dependences of *AP* on the reaction time demonstrated that the formation of the *AP* complex reached equilibrium by the time the liquid reached the analytic zone (*t*–*t*_1_) only at *k_a_*_1_ = 10^6^ M^−1^·s^−1^. For example, taking [*A*]_0_ = 10^−5^ M, [*P*]_0_ = 10^−7^ M, *x* = 0.0001, we can find that the formation of 90% from the maximal value of *AP* complexes takes 22 s at *k_a_*_1_ = 10^6^ M^−1^·s^−1^, 230 s at *k_a_*_1_ = 10^5^ M^−1^·s^−1^ and 2320 s at *k_a_*_1_ = 10^4^ M^−1^·s^−1^. This result indicated that the reactions in the studied immunochromatographic system usually occurred under nonequilibrium conditions.

### 3.3. Consideration of the Model: Stage 2

The molecules of the antigen in the analytic zone reacted with immunoglobulins according to Equations (2) and (3). Accordingly, the total rate of change in [*R*] can be expressed by the following formula (the value of *k_d_* is neglected according to condition #6 (see Section 3.1):

(7) $$-\frac{\partial \left[R\right]}{\partial t_{1}}\;=k_{a2}\left[A\right]\left[R\right]+k_{a3}\left[AP\right]\left[R\right]$$

Taking into account the conditions *k_a_*_2_ ≈ *k_a_*_3_ and [*A*] + [*AP*] = [*A*]_0_, Equation (6) can be simplified to

(8) $$-\frac{\partial \left[R\right]}{\partial t_{1}}=k_{a3}{\left[A\right]}_{0}\left[R\right]$$

Thus, the [*R*] value may be determined as follows:

(9) $$\left[R\right]={\left[R\right]}_{0}\;e^{-k_{a3}\left[A_{0}\right]t_{1}}$$

The interaction of *R* with immunoglobulins bound and not bound to *P* leads to the formation of *AR* and *APR* complexes. Accordingly,

(10) $$\left[AR\right]={\left[R\right]}_{0}-\left[R\right]-\left[APR\right]$$

The initial concentration of IgG (i.e., the main class of immunoglobulins) in sample [A]_0_, for example, in human serum was 6–20 mg/mL (or 4 × 10^−5^–10^−4^ M) [22]. The concentration of IgG against individual antigens in human blood did not exceed 50 μg/mL, which was demonstrated in [23,24,25,26]. Thus, the x value in this model was less than 0.01. It follows that most AR complexes contained immunoglobulins that are not specific to the antigen used and are incapable of forming the APR complex. Only a small part of the AR complexes contained specific antibodies (AR_sp_) and interacted with P in reaction (4) or had already formed the AP complex and reacted with R during the reaction (3). To calculate the rate of the APR formation, it is necessary to establish how [AR]_sp_ changes with time.

If *P* is excluded from this system and the sample is passed without the marker conjugate through the system, only the reaction of free immunoglobulins (A) with the antigen-receptor (*R*) will occur in the analytic zone. In this case, [*AR*] = [*R*]_0_ − [*R*] and, accordingly, [*AR*]*_sp_* = ([*R*]_0_ − [*R*]) *x*. If the component *P* is added, then in the analytic zone, in addition to the *AR* complex, the *APR* complex will be formed. Hence,

(11) $${\left[AR\right]}_{sp}=\left({\left[R\right]}_{0}-\left[R\right]\right)x-\left[APR\right]$$

The *APR* complex can be formed by reactions (3) and (4). Accordingly, the [*APR*]_3_ and [*APR*]_4_ values can be separated as the concentrations of complexes formed by these methods such that ([*APR*] = [*APR*]_3_ + [*APR*]_4_). The rates (*v*) in these changes in concentration are described by the following equations:

(12) $$\frac{\partial {\left[APR\right]}_{3}}{\partial t_{1}}=k_{a3}\left[AP\right]\left[R\right]$$

(13) $$\frac{\partial {\left[APR\right]}_{4}}{\partial t_{1}}\;=k_{a4}{\left[AR\right]}_{sp}\left[P\right]$$

The rate of [*AR*]*_sp_* change:

(14) $$\frac{\partial {\left[AR\right]}_{sp}}{\partial t_{1}}\;=k_{a3}{\left[A\right]}_{sp}\left[R\right]-\frac{\partial {\left[APR\right]}_{4}}{\partial t_{1}}$$

Substituting expression (13) and the expressions [*A*]*_sp_* = *x·*[*A*]_0_ − [*AP*], [*P*] = [*P*]_0_ − [*AP*] into the Equation (14), results in the following equation:

(15) $$\frac{\partial {\left[AR\right]}_{sp}}{\partial t_{1}}=k_{a3}\left(x{\left[A\right]}_{0}-\left[AP\right]\right)\left[R\right]-k_{a4}{\left[AR\right]}_{sp}\left({\left[P\right]}_{0}-\left[AP\right]\right)$$

Because the time dependence of [*AP*] is known (see Equation (6)), Equation (15) contains only one unknown function, [*AR*]*_sp_*, and is a linear, first-order differential equation. Solving this equation (see the Appendix A) results in the following function:

(16) $${\left[AR\right]}_{sp}=\frac{x\left[R_{0}\right]\left(\left[P_{0}\right]-\left[A_{0}\right]x\right)\left(1-\;e^{-t_{1}k_{a3}\left[A_{0}\right]}\right)}{\left[P_{0}\right]e^{tk_{a1}m}-\left[A_{0}\right]x}$$

The function of the dependence of [*APR*] on time is found in Equations (11) and (16):

(17) $$\left[APR\right]=\frac{x\left[R_{0}\right]\left[P_{0}\right]\left(1-e^{-t_{1}k_{a3}\left[A_{0}\right]}\right)\left(e^{tk_{a1}\left(\left[P_{0}\right]-\left[A_{0}\right]x\right)}-1\right)}{\left[P_{0}\right]e^{tk_{a1}\left(\left[P_{0}\right]-\left[A_{0}\right]x\right)}-\left[A_{0}\right]x}$$

Using expression (5), Equation (15) can be represented as follows:

(18) $$\left[APR\right]=\frac{\left[R_{0}\right]\left[AP\right]}{\;\left[A_{0}\right]}\left(1-e^{-t_{1}k_{a3}\left[A_{0}\right]}\right)$$

As noted in Section 3.1, the main task in constructing a model is describing the *APR* complex kinetics. Therefore, Equation (18) is the desired solution. For a fixed time *t*_1_ and varied concentration [*A*]_0_, this equation best described the behavior of the calibration dependence of the analysis.

### 3.4. Theoretical Comparison of 2 Schemes of Immunochromatographic Serodiagnosis

In the serodiagnostic ICA, both schemes A and B included the reactions according to Equations (1)–(4). The difference is that in scheme B, only specific antibodies participated in reactions (1) and (4), whereas in scheme A, all immunoglobulins were involved in these reactions. Moreover, in scheme B, all immunoglobulins were involved in reactions (2) and (3), whereas in scheme A, only specific antibodies took part in these reactions.

The comparison of the schemes is based on Equation (18) for scheme B and the corresponding equation for scheme A, which was derived in [17]:

(19) $$\left[APR^{\prime }\right]=\frac{k_{a3}^{\prime }{\left[P^{\prime }\right]}_{0}{\left[R^{\prime }\right]}_{0}}{k_{a2}^{\prime }{\left[A^{\prime }\right]}_{0}}\;\left(1-e^{-k_{a2}^{\prime }x{\left[A^{\prime }\right]}_{0}t_{1}}\right)$$

where *k*′*_a_*_2_, *k*′*_a_*_3_, *k*′*_a_*_2_ and *k*′*_a_*_3_, respectively, are the binding constants of the free and labeled specific antibodies to the antigen immobilized in the analytic zone; *A*′ is the immunoglobulin molecules in the sample; *P*′ is the active molecules of immunoglobulin-binding protein immobilized on a gold nanoparticle; *R*′ is the active antigen molecules immobilized in the analytic zone; and *APR*′ is the detectable complex formed in the analytic zone. Other designations are identical to those used for scheme B.

**Figure 3:**
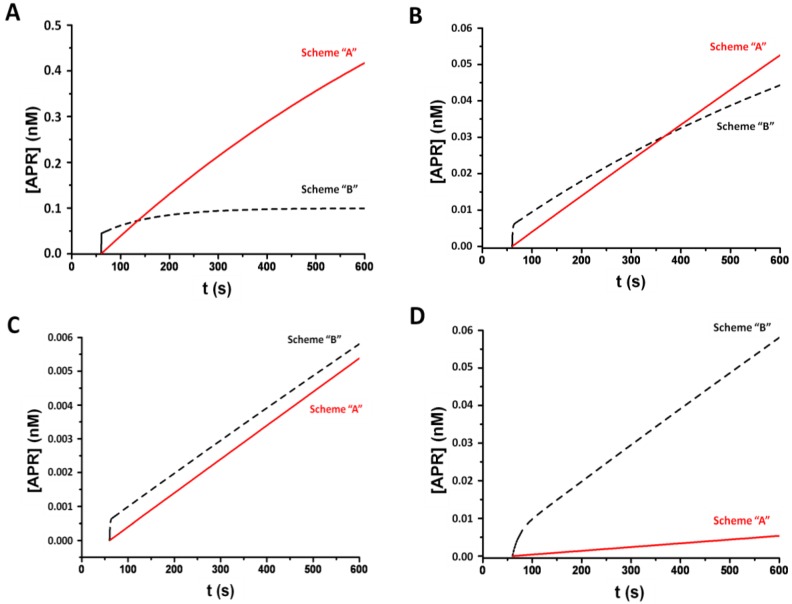
Kinetics of the *APR* formation in schemes A and B of the ICA; the model parameters are as follows: [A]_0_ = 10^−5^ M, [P]_0_ = 10^−7^ M, *x* = 10^−4^; t–t_1_ = 60 s. (**A**) k_a1–4_ = 10^6^ M^−1^·s^−1^, [R]_0_ = 10^−6^ M; (**B**) k_a1−4_ = 10^5^ M^−1^·s^−1^, [R]_0_ = 10^−6^ M; (**C**) k_a1–4_ = 10^4^ M^−1^·s^−1^, [R]_0_ = 10^−6^ M; (**D**) k_a1−4_ = 10^4^ M^−1^·s^−1^, [R]_0_ = 10^−6^ M in scheme A and [R]_0_ = 10^−5^ M in scheme B.

The [*APR*] changes for the two serodiagnostic ICA schemes were plotted by varying the kinetic association constants in the 10^4^–10^6^ M^−1^·s^−1^ range of values, which is typical in antigen–antibody interactions (see Figure 3). The concentrations of reagents typical for serodiagnostic ICA were used.

Figure 3A shows that, in principle, schemes A and B reacted differently to the change in the associated antigen–antibody constant. At the binding constant 10^6^ M^−1^·s^−1^, the initial rate of *APR* formation was higher in t scheme B than in scheme A. However, the rate of *APR* formation in scheme B decreased, whereas in scheme A, it remained almost unchanged during the entire reaction time in the analytic zone. Consequently, at 1 min after the start of the reaction in the analytic zone, the signal in scheme A was higher than in scheme B for the given model parameters.

A similar situation was observed in *k_a_* = 10^5^ M^−1^·s^−1^, but the signal in scheme A exceeded the signal in scheme B only at 5 min after the start of the reaction in the analytic zone (Figure 3B). When the association constant was lowered by another order of magnitude, the [*APR*] value for scheme B was higher than for scheme A throughout the analysis time (Figure 3C). Thus, scheme B allowed more effective detection of low-affinity antibodies (*k_a_* below 10^4^ M^−1^·s^−1^) than scheme A did at identical reagent concentrations.

Scheme B allowed for increasing the concentration of immunoglobulin-binding protein values comparable to the concentration of immunoglobulins in the blood. This result can be explained by the fact that the immunoglobulin-binding protein was immobilized on a nitrocellulose membrane that has a limiting sorption capacity of up to 15 mg of protein per 1 mL of internal membrane volume, which is in accordance with 5 μg of protein in the analytic zone. In scheme A, the immunoglobulin-binding protein was immobilized on gold nanoparticles, and the sorption capacity was several orders of magnitude lower than the capacity of the nitrocellulose membrane [17]. Therefore, the concentration of the immunoglobulin-binding protein in the gold nanoparticle conjugate was only up to 100 μg/mL. By taking into account the volume of the conjugate used in ICA, about 5 μL, it was possible to bind no more than 0.5 μg of the immunoglobulins in the sample. Therefore, in scheme B, when saturating concentrations of immunoglobulin-binding protein were used, the binding capacity of the immunoglobulins in the sample was much higher than in scheme A.

The kinetics of the *APR* formation with the tenfold increase in the concentration of [*R*]_0_ = 10^−5^ M in comparison with the kinetics of the *APR* formation in scheme A at [*R*]_0_ = 10^−6^ M (Figure 3D) demonstrated the possibility of increasing the signal by using the saturating concentration of the immunoglobulin-binding protein. The kinetic binding constant for antibodies is usually much lower than 10^6^ M^−1^·s^−1^ [20]. Therefore, the sensitivity of the antibody detection in scheme A in most cases should be lower than in scheme B. In addition, the concentration of the immunoglobulin-binding protein in scheme B may be increased by an order of magnitude. Accordingly, scheme B allowed for the inclusion of more antibodies in the detectable *APR* complex.

### 3.5. Testing Patients’ Serum Using ELISA

**Figure 4:**
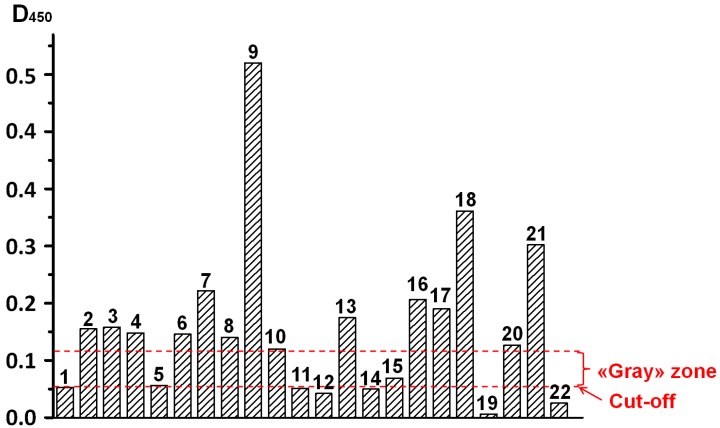
Results of ELISA serodiagnostic of serum from patients with human pulmonary tuberculosis; sera dilution: 100 times.

A panel of 22 sera from patients with tuberculosis of the respiratory system was tested by ELISA to determine the content of antibodies against mycobacterial antigen Rv0934. The assay for each sample was implemented in triplicates, and average optical density is further considered. The OD_450_ value of the substrate solution was used as the characteristic of the antibody content. The results of the test are shown in Figure 4. As a negative control, 10 sera from healthy donors were used. The maximal ELISA staining for sera from the healthy donors (OD_450_ = 0.055) was the threshold (i.e., cut-off) level. Sera with an ELISA signal that exceeded the threshold level by less than 2 times were considered questionably positive samples. Sera with an ELISA signal that exceeded the threshold level more than 2 times were considered positive samples. Based on the results of the ELISA serodiagnostic testing, 14 of 22 sera (64%) were classified as positive, and another two sera yielded intermediate results.

### 3.6. Experimental Comparison of 2 Schemes of Immunochromatographic Serodiagnosis

**Figure 5:**
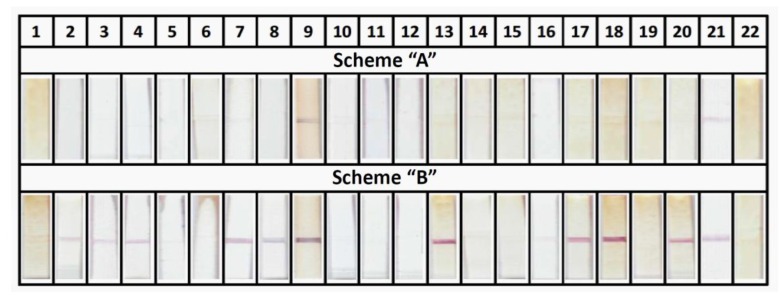
Tested sera of tuberculosis patients by schemes A and B of immunochromatographic serodiagnostics.

The prepared experimental samples of the test strips (both schemes A and B) were tested on serum samples from patients with respiratory tuberculosis (22 sera) and healthy donors (10 sera). The conclusion about respiratory tuberculosis was made by Dr. V. G. Avdienko (Central Tuberculosis Research Institute, Moscow, Russia) basing on bacteriological testing and a combination of symptoms. The assay for each sample was implemented in triplicates. The use of the test strips in scheme A showed extremely low diagnostic efficiency: only 2 of 22 sera from patients with a diagnosis of tuberculosis yielded positive results. When the tests strips in scheme B were applied, 12 of 22 sera were characterized as positive (55%), including 2 sera that were positive according to scheme A. In the analysis of sera from 10 healthy donors, no false positive test results were observed in either scheme. Thus, if ELISA is considered the reference technique, the sensitivity of ICA scheme B for the studied set of samples is 86%, and the specificity is 100%. Figure 5 illustrates the appearance of the strips (fragments of analytic zone) after the analysis of sera from patients with respiratory tuberculosis. Table 1 shows a summary of the results of the ELISA and ICA sera testing.

Thus, in comparison to scheme A, scheme B improved the detection of patients with pulmonary tuberculosis by 6 times. This result supported the theoretical predictions, which were obtained based on the analysis of the models of the 2 schemes. The ICA system based on scheme B approached the ELISA method according to the diagnostic sensitivity, but it surpassed ELISA in in terms of diagnostic speed; the immunochromatography results were obtained in 10 min.

## 4. Conclusions

A new model of immunochromatographic serodiagnosis was proposed for a scheme in which a labeled nanoparticle was conjugated with the antigen of a pathogen, and the immunoglobulin-binding protein was adsorbed in the analytic zone of the test (scheme B). The model was analyzed using an uncountable approach, which made it possible to derive the general kinetic equation for the formation of a detected complex in the analytic zone. The model was used for theoretical assessment of the influence of several factors (e.g., reagent concentrations, interaction constants, and duration of analysis stages) on the results of the analysis, and it could be a useful tool for the developers of immunochromatographic tests. Understanding the patterns of immune complexes formation in rapid immunochemical tests will contribute to the effective development of immunoassay systems belonging to new generation, including various formats of microfluidic and microchip immunoassays [27].

The results showed that scheme B theoretically allowed for more efficient detection of low-affinity antibodies in the sample (*k_a_* < 10^5^ M^−1^·s^−1^) than the widely used scheme A did in the immunochromatographic serodiagnosis, in which gold nanoparticles were conjugated with an immunoglobulin-binding protein and the antigen was adsorbed in the analytic zone of the test. Using the example of the ICA of specific antibodies against the Rv0934 antigen of *M. tuberculosis*, the experimental results demonstrated the possibility of increasing the diagnostic sensitivity of similar analyses using scheme B instead of scheme A.

## Figures and Tables

**Table 1:** Results of testing sera from patients with respiratory tuberculosis using ELISA and the 2 ICA schemes (average values for triplicate measurements).

Serum Number	ELISA		ICA, Scheme A		ICA, Scheme B	
	OD_450_	Qualitative Result	Color Intensity *	Qualitative Result	Color Intensity *	Qualitative Result
1	0.053	–	0	–	0.5	–
2	0.155	+	0	–	2.2	+
3	0.158	+	0	–	1.9	+
4	0.148	+	0	–	3.4	+
5	0.056	±	0	–	0.5	–
6	0.146	+	0.2	–	0.4	–
7	0.222	+	0.1	–	6.8	+
8	0.140	+	0	–	4.1	+
9	0.620	+	3.9	+	11.0	+
10	0.120	+	0.2	–	0.3	–
11	0.051	–	0.1	–	0	–
12	0.042	–	0	–	0	–
13	0.175	+	0.2	–	8.5	+
14	0.050	–	0	–	0	–
15	0.069	±	0.2	–	0	–
16	0.206	+	0	–	0.7	+
17	0.190	+	0.1	–	7.7	+
18	0.361	+	0	–	12.1	+
19	0.006	–	0	–	0	–
20	0.127	+	0	–	6.5	+
21	0.302	+	2.4	+	4.3	+
22	0.025	–	0.5	–	0.3	–

* Relative units of Reflekom measurements.

## Appendix A. The Solution of the Differential Equation (15)

The expression [*AP*] (6) is substituted into Equation (15). The following notations are then introduced: [*AR*]*_sp_* = *y* and [*P*]_0_ – *x*·[*A*]_0_ = *m*. Then, after arithmetic simplifications, the Equation (15) is transformed into the following:

(A1)
$$\frac{\partial y}{\partial t_{1}}+\frac{k_{a4}\left[P_{0}\right]me^{tk_{a1}m}}{\left[P_{0}\right]e^{tk_{a1}m}-\left[A_{0}\right]x}y-\frac{k_{a3}x\left[A_{0}\right]\left[R_{0}\right]me^{-t_{1}k_{a3}\left[A_{0}\right]}}{\left[P_{0}\right]e^{tk_{a1}m}-\left[A_{0}\right]x}=0$$

This equation is solved using the Bernoulli method as follows:

The y function is represented as the composition (i.e., multiplication) of two functions—*u* and *v*: $y=uv,\;\frac{\partial y}{\partial t_{1}}=\frac{\partial u}{\partial t_{1}}v+\frac{\partial v}{\partial t_{1}}u$ . The transformed *y* is substituted into the solved differential equation:

(A2)
$$\frac{\partial u}{\partial t_{1}}v+\frac{\partial v}{\partial t_{1}}u+\frac{k_{a4}\left[P_{0}\right]me^{tk_{a1}m}}{\left[P_{0}\right]e^{tk_{a1}m}-\left[A_{0}\right]x}uv-\frac{k_{a3}x\left[A_{0}\right]\left[R_{0}\right]me^{-t_{1}k_{a3}\left[A_{0}\right]}}{\left[P_{0}\right]e^{tk_{a1}m}-\left[A_{0}\right]x}=0$$

The following system of equations is then created:

{(A3)∂v∂t1+ka4[P0]metka1m[P0]etka1m−[A0]xv=0(A4)∂u∂t1v−ka3x[A0][R0]me−t1ka3[A0][P0]etka1m−[A0]x=0

The Equation (A3) is then solved. The variables can be separated as follows:

(A5)
$$\frac{\partial v}{v}=-\frac{k_{a4}\left[P_{0}\right]me^{tk_{a1}m}}{\left[P_{0}\right]e^{tk_{a1}m}-\left[A_{0}\right]x}\partial t_{1}$$

Next, both sides of the Equation (A4) can be integrated as follows:

(A6)
$$\ln\left(v\right)=-k_{a4}\left[P_{0}\right]m\;\int \frac{e^{tk_{a1}m}}{\left[P_{0}\right]e^{tk_{a1}m}-\left[A_{0}\right]x}\partial t_{1}$$

Note that $\partial t_{1}=\partial t$ because *t* and *t*_1_ differ by a constant.

An additional parameter, *z*, is then proposed: $\;\left[P_{0}\right]e^{tk_{a1}m}-\left[A_{0}\right]x=z$ , then $\frac{\partial z}{\partial t}=k_{a1}\left[P_{0}\right]me^{tk_{a1}m}$ . This notation allows rewriting the Equation (A6) as the following:

(A7)
$$\ln\left(v\right)=-\frac{k_{a4}}{k_{a1}}\int \frac{\partial z}{z}$$

Using the statement that *k_a_*_1_ ≈ *k_a_*_4_, the integral on the right side of the Equation (A5) can be taken as

(A8)
ln(v)=−ln(z)

Hence, *v = 1/z*.

Equation (A4) can then be solved and rewritten using the introduced notations:

(A9)
$$\frac{\partial u}{\partial t_{1}}=\frac{k_{a3}x\left[A_{0}\right]\left[R_{0}\right]me^{-t_{1}k_{a3}\left[A_{0}\right]}}{zv}$$

Since *v = 1/z*, then:

(A10)
$$\frac{\partial u}{\partial t_{1}}=k_{a3}x\left[A_{0}\right]\left[R_{0}\right]me^{-t_{1}k_{a3}\left[A_{0}\right]}$$

Both sides of the Equation (A10) are then integrated:

(A11)
$$u=k_{a3}x\left[A_{0}\right]\left[R_{0}\right]m\int e^{-t_{1}k_{a3}\left[A_{0}\right]}\partial t_{1}$$

(A12)
$$u=-x\left[R_{0}\right]me^{-t_{1}k_{a3}\left[A_{0}\right]}+Const$$

Returning to the initial notations:

(A13)
$${\left[\mathrm{AR}\right]}_{\mathrm{sp}}=\frac{Const-x\left[R_{0}\right]m\;e^{-t_{1}k_{a3}\left[A_{0}\right]}}{\left[P_{0}\right]e^{tk_{a1}m}-\left[A_{0}\right]x}$$

Const is found from the boundary conditions: for *t*_1_ = 0, [*AR*]*_sp_* = 0.

(A14)
$$Const=x\left[R_{0}\right]\left(\left[P_{0}\right]-\left[A_{0}\right]x\right)$$

Then:

(A15)
$${\left[\mathrm{AR}\right]}_{\mathrm{sp}}=\frac{x\left[R_{0}\right]\left(\left[P_{0}\right]-\left[A_{0}\right]x\right)\left(1-e^{-t_{1}k_{a3}\left[A_{0}\right]}\right)}{\left[P_{0}\right]e^{tk_{a1}m}-\left[A_{0}\right]x}$$
